# Healthcare quality and safety assessment based on annual scorekeeping

**DOI:** 10.3389/fpubh.2022.937338

**Published:** 2022-09-09

**Authors:** Guo-Mo Liang, Wen-Chao Xie, Mei Gan, Jiao-Wei Gao, Qing Liang, Zhi-Yu Zeng

**Affiliations:** ^1^Department of Quality Control, The Sixth Affiliated Hospital of Guangxi Medical University, Yulin, China; ^2^The Sixth Affiliated Hospital of Guangxi Medical University, Yulin, China; ^3^Department of Medical, The Sixth Affiliated Hospital of Guangxi Medical University, Yulin, China; ^4^Guangxi Medical University, Nanning, China

**Keywords:** annual scorekeeping, quality management, assessment system, healthcare quality, healthcare safety

## Abstract

**Objective:**

To explore the practice of medical quality and safety evaluation system based on annual score under the background of establishing modern hospital management system and strengthening national public hospital performance evaluation.

**Methods:**

Statistical analysis was used to study the improvement of medical quality and safety in hospitals after the implementation of score evaluation, and the existing problems were analyzed according to the actual situation and related requirements.

**Results:**

The hospital's medical quality and safety evaluation system ran smoothly, the evaluation indexes could be implemented, and the evaluation results were used properly. The improvement of hospital medical quality and operation efficiency has achieved good results.

**Conclusion:**

The evaluation system of medical quality and safety for physicians and medical technicians based on annual score can achieve the whole process, all-round, personalized and information-based evaluation, and promote the high-quality development of hospitals. It is necessary to further improve the range of evaluation and carry out the evaluation of the evaluation system by relevant personnel.

The provision of high-quality healthcare is fundamental to the survival and development of a hospital, and, thus, it is the main focus of hospital management (1). China has made great progress in healthcare, but there are still gaps in the quality of healthcare, with most process indicators needing improvement (2). The government has recently issued the following guidelines: Measures for Healthcare Quality Management; Guidance on Establishing a Modern Hospital Management System; and Opinions on Strengthening the Performance Appraisal of Tertiary Public Hospitals. These documents suggest that the hospital management model should change from a crude one to a more refined one, and they guide tertiary public hospitals in strengthening their healthcare quality management and patient safety management while implementing functional positioning. Therefore, based on the concept of a “driver's license” in traffic management, the “scorekeeping system” assessment was introduced to healthcare quality management (3). An annual scorekeeping assessment system was established in the hospital for doctors as well as other medical and technical staff to improve healthcare quality and safety. It aimed to standardize the medical service, improve the overall quality of medical personnel and medical technology, eliminate medical defects, errors, and accidents, reduce the incidence of medical disputes, protect medical safety, and safeguard healthcare rights.

## The establishment of the assessment system

A long-term supervision mechanism for assessing healthcare quality and safety was established, using quantitative scorekeeping records and an incentive and penalty assessment system, with the participation of all staff. Their enthusiasm was generated by the improvement of the internal staff performance appraisal system, as well as the scientific and reasonable distribution of performance (4, 5). The development of a strong quality healthcare culture and the implementation of the patient-centered service concept, combining concern for patient health with the long-term development of the hospital, aimed to improve the overall quality of healthcare and ensure medical safety.

## The main content of the assessment system

### System design

The main structure of the system included the deduction section and the incentive one, and the assessment methods were divided into three types according to the characteristics of different specialties. There were those assessing the staff in the inpatient clinical departments, those evaluating the staff in the non-inpatient clinical departments, and those assessing the medical and technical department staff. However, the characteristics of all medical staff were combined with an emphasis on the people-oriented context of the assessment indicators and scorekeeping (6).

### The assessment indicators and scorekeeping without a cap

#### The penalty scorekeeping section

This section had two major components, namely, the public assessment and the specialist assessment, with 44 primary indicators and 295 secondary indicators. The important primary indicators in the public assessment program covered seven areas as follows: the practice of medicine according to law; medical record writing and the medical scoring system; rational examination (medication, treatment, use of high-value consumables); case management; nosocomial infection management; medical insurance service management; and medical ethics and medical spirits management. The specialist assessment covered anesthesia, pharmacy, blood transfusion, medical imaging, medical laboratory, and pathology (Table 1).

**Table 1:** The indicators in the annual scorekeeping assessment system for healthcare quality and safety (The penalty section in scorekeeping).

**The primary indicator**	**The secondary indicator**	**Score in the scorekeeping**
**Some items in the public assessment**		
1 The practice of medicine according to law	1 Independent on the duty of the un-registered medical technical staff	2 points/time
	2 Treatment activities beyond the scope of the license registration	4 points/time
2 Medical record writing and medical score system,	1 Improper writing of medical records	0.5–1 point/time
	2 Without rectification of the defective medical record within 3 days after notification	0.5 point/time
	3 Violation of medical score system	1–4 points/time
	4 With <2/3 of the correct answer to the questions targeting the core system	0.5 point/time
3 Rational examination (medication, treatment, application of high-value consumables)	1 Examination without indication	1 point/time
	2 Improper medication	1 point/time
	3 Application of high-value consumables without indication	4 point/time
4 Case management	1 Failure to submit the final medical records within seven workdays after discharge	0.3 point/copy/day
	2 Failure to submit the medical records of patient discharged in the previous month for more than 10 days	0.5 point/copy/day
5 Nosocomial infection	1 Underreporting of the case with nosocomial infection	1 point/time
	2 Inadequate implementation of disinfection and isolation of patients with multi-drug resistant bacteria	1 point/time
	3 Unqualified hand hygiene	1 point/time
6 Medical insurance service management	1 Failure to conduct the hospitalization and discharge criteria, with bed hanging and disaggregation of hospitalization	1 point/time
	3 Unqualified hand hygiene	1 point/time
7 Medical ethics and medical spirits management	1 Violation of the healthcare “nine forbidden”	20–30 points/time
	2 Bad influence as a result of bad service attitude	4–10 points/time
**Some items in the specialty assessment**		
1 Anesthesiology	1 Over-privilege operation	1 point/time
	2 Failure of conduction of pre-anesthesia discussion as needed	1 point/time
2 Department of pharmacy	1 Dispensing medication without effective intervention for abnormal prescriptions	1 point/time
	2 Sending the wrong medicine	1 point/time
3 Blood transfusion division	1 Failure to check for verification with the blood receiver as required	2 point/time
	2 Failure to complete the pre-transfusion testing program as required	4 point/time
4 Medical imaging	1 Wrong, inaccurate and improper report	1–4 point/time
	2 Failure to implement the “Critical Values” reporting system	4 point/time
5 Medical laboratory	1 Wrong, inaccurate and improper report	1–4 point/time
	2 Results were not reviewed in doubt and not reported to clinical departments promptly	1 point/time
6 Pathology	1 Wrong, inaccurate and improper report	1–4 point/time
	2 Loss of the pathological slice but without adverse consequence	1 point/time

#### The incentive scorekeeping section

This section was composed of nine primary indicators as follows: paper publication, scientific research proposals, scientific research awards, honorary titles, news reports, teaching, new technology and innovative projects, internal competition, and talent cultivation. There were also 12 secondary indicators (Table 2).

**Table 2:** The indicators in the annual scorekeeping assessment system for healthcare quality and safety (The incentive section in scorekeeping).

**The primary indicator**	**The secondary indicator**	**Score in the scorekeeping**
1 Paper publication	1 Publication of the Chinese core, Chinese series, and SCI articles	4–8 points/piece
2 Scientific research proposal	1 Obtaining the provincial and national research projects	6–10 points/project
3 Scientific research award	1 Obtaining the local, provincial and national research awards	3–15 points
4 Honorary title	1 Obtaining local, provincial, and national honorary title	3–10 points
5 News report	1 Positive news report by the municipal and above media	3 points
6 Teaching	1 Excellent in the overall evaluation of teaching quality assessment	2 points
	2 Teaching secretary with excellent evaluation by the competent department	3 points
7 New technology and new project	1 Conduction and achievement of the expected economic and social benefits as planned	3 points
8 Internal competition	1 Obtaining various rewards in medical-related competitions	2 points
9 Talent cultivation	1 Obtaining a mentorship for the postgraduate	80 points
	2 Successful establishment of a master's degree awarding site in the department	50–100 points
	3 With on MD or PD cultivated in the department	15–50 points

## Implementation of the one-year assessment cycle

### Assessment departments and procedures

Led by the Medical Department, each department was responsible for the monthly assessment, and the results were entered into the medical information management system and publicized before the 10th of the following month (appeals had to be made within seven days, if there were any objections, and reported to the Healthcare Quality and Safety Management Committee for discussion and review). The results were announced in the office automation system, the medical information management system, or at a middle-level management meeting, and, finally, they were summarized with other quality management indicators by the Department of Quality Control and reported to the Finance Department (the Performance Office) for deduction and penalty.

### Assessment results

As is shown in Table 3, the results were divided into three categories indicating a pass, a borderline score (necessitating a warning), and a failure. The borderlines were determined according to the three types of medical staff, namely, those in the inpatient clinical departments, those in non-inpatient clinical departments, and those in the medical and technical departments.

**Table 3:** The hierarchy of annual scorekeeping assessment results for doctors, medical and technical staff.

**Category**	**Pass**	**Warning**	**Failure**
The inpatient clinical departments	≤60 points	60–72 points	>72 points
The non-inpatient clinical departments	≤24 points	24–36 points	>36 points
The medical and technical departments	≤12 points	12–18 points	>18 points

### Application of assessment results

Penalties: (1) A warning involved the cancellation of the awarding of any merit, priority, or honorary title, regardless of level, during the year in question and a one-time performance penalty of 30 points at the end of the year (only with deductions and penalties, without scorekeeping), and department rectification was required; (2) A failure meant that in addition to the penalties in the warning section, a job suspension was implemented for 3 months while training was undertaken, and reemployment was at a lower technical or medical grade for 1 year. In addition, applying for promotion the following year was prohibited, and department rectification was required.

Deductions and performance penalties were as follows: (1) personal performance was linked to the results of the monthly individual scorekeeping assessment. One penalty point was equal to 100 yuan, and the appropriate amount was directly deducted from the monthly salary (the deductions of inpatient trainee staff were capped at a limit of 500 yuan); (2) The individual monthly scorekeeping was also linked to the department performance, and the individual was penalized for the total performance score of the department; (3) Performance of the department director: if the average scorekeeping of individuals (apart from the director and deputy director) in the department was >3 points over the month, the director and deputy director would score three and two points, respectively, and the performance would be deducted from their salaries. If two or more staff failed during a particular year, the director and deputy director would score 10 and 8 points, respectively, and the performance would be deducted from their december salary.

#### Incentive scorekeeping

The incentive scores were totaled, and individual rewards were received at the end of the year, with one performance point equal to 100 yuan. If the incentive scorekeeping in the year in question did not achieve a pass, that is to say, those in the inpatient clinical departments, non-inpatient clinical departments, and medical and technical departments obtained scores ≤48, 24, and 12 points, respectively, they could apply for a delay in the imposition of any penalties for 1 year while they were closely monitored. If the scorekeeping was ≤36, 18, and 9 points in the following year, the penalty could be canceled. Otherwise, the penalty of the previous year would be incurred. For those with no pass in the following year, the penalty would be imposed for two consecutive years, along with a ban on applying for a further technical title within the following 3-year period.

## The effects of assessment

After nearly two annual scorekeeping cycles, there were noticeable improvements in the hospital. The quality of medical care and safety management had been strengthened as a result of the increased awareness of professional ethics, discipline, and responsibility among the medical personnel. Medical treatment had been further standardized with the optimization of the service connotations, the implementation of patient safety goals, and improvements to medical risk prevention. The operational efficiency and performance assessment scores of the hospital had also been greatly improved. The average length of one hospital stay in 2021 was shortened by 1.25 days compared with the previous year, and the performance assessment scores of provincial tertiary public hospitals had increased by 0.98 points. Score comparison between 2021 and 2020: the average score of doctors and medical technicians decreased by 0.01 points per month, as shown in Table 4. The monthly average score of medical record management, medical record writing, medical score system and medical ethics management of public assessment items decreased, while reasonable examination (medication, treatment and use of high-value consumables), management requirements of other links and nosocomial infection management increased, as shown in Table 5. The average monthly per capital score of clinical departments in the inpatient department decreased in 18 departments, such as Gastrointestinal Gland Surgery, Neurosurgery, Spine Surgery, etc., while increased in 15 departments, such as Neurology, Nephrology, Respiratory Medicine, etc., while remained unchanged in Oncology Department, as shown in Table 6. The average monthly per capital score of non-inpatient clinical departments, medical technology departments decreased in 7 departments, such as Pediatric Rehabilitation Medicine, the Third Outpatient, Emergency Department, etc., while increased in Medical Cosmetology, Pharmacy Department and Nutritional Department. There were no changes in Anesthesiology and Clinical Laboratory. And Emergency Center, Ultrasonography lab, Physical Examination Center, Electrocardiogram, Pathology Department etc. remained 0 in 2 years, shown in Table 7. The monthly average scores of medical record department, Quality Control Department, Outpatient Department, Pharmacy Department and Blood Transfusion Department decreased, while the monthly average scores of Department of Medical Administration, Prevention and Health Care Department and infection Management Department increased, as shown in Table 8. In 2021, four inpatient clinical department personnel were punished, as shown in Table 9.

**Table 4:** The hospital scored per capital monthly in 2020 and 2021 (scores).

**Category**	**2020**	**2021**
The inpatient clinical departments	0.62	0.61
The non-inpatient clinical departments	0.08	0.08
The medical and technical departments	0.02	0.01
Hospital-wide	0.24	0.23

**Table 5:** The monthly average score of public assessment items in 2020 and 2021 (scores).

**Public assessment items**	**2020**	**2021**
Medical record management	109.72	105.88
Medical record writing and medical score system	91.25	85.33
Reasonable examination (medication, treatment and use of high-value consumables)	10.33	13.67
Medical ethics management	19.17	12.50
Management requirements of other links	3.83	6.00
Nosocomial infection management	1.75	3.75

**Table 6:** The average monthly per capital score of clinical departments in the inpatient department in 2020 and 2021 (scores).

**Department**	**2020**	**2021**	**Department**	**2020**	**2021**
Neurology	0.53	1.22	Obstetric	0.05	0.03
Nephrology	0.28	0.90	Intensive care	0.28	0.26
Respiratory medicine	0.38	0.88	Pediatric surgery	0.13	0.09
Cardiology	0.57	0.94	Neonatology	0.26	0.21
Bone and joint sports surgery	1.84	2.13	Hepatological surgery	1.03	0.98
Infectious diseases	0.33	0.62	Bone trauma hand surgery	1.18	1.08
Ophthalmology	0.70	0.97	Rheumatology and immunology	0.27	0.16
Digestive internal medicine	0.18	0.37	Orthopedics	0.48	0.29
Colorectal surgery	0.61	0.77	Endocrinology	0.35	0.11
Otolaryngology	1.01	1.15	Intervention	0.36	0.11
Blood internal medicine	0.09	0.23	Traditional Chinese medicine	0.59	0.32
Pediatric medicine	0.35	0.47	Gynecology	0.59	0.28
General practice	0.27	0.36	Urinary surgery	0.71	0.40
Geriatrics	0.38	0.47	Cardiothoracic vascular surgery	2.37	2.01
Rehabilitation medicine	0.14	0.19	Spine surgery	0.71	0.26
Oncology	0.31	0.31	Neurosurgery	1.76	0.93
Stomatology	0.14	0.13	Gastrointestinal gland surgery	1.89	0.50

**Table 7:** The average monthly per capital score of non-inpatient clinical departments, medical technology departments in 2020 and 2021 (scores).

**Non-inpatient clinical departments**	**2020**	**2021**	**Medical technology departments**	**2020**	**2021**
Emergency center	0.00	0.00	Ultrasonography lab	0.00	0.00
Physical examination center	0.00	0.00	Electrocardiogram	0.00	0.00
Medical cosmetology	0.00	0.03	Pathology department	0.00	0.00
Anesthesiology	0.01	0.01	Blood transfusion department	0.00	0.00
Pediatric rehabilitation medicine	0.03	0.00	Pharmacy department	0.00	0.05
The third outpatient	0.04	0.00	Nutritional department	0.00	0.21
Outpatient department	0.08	0.05	Clinical laboratory	0.03	0.03
Emergency department	0.25	0.09	Nuclear medicine department	0.05	0.02
Dermatology department	0.25	0.15	Radiology department	0.10	0.00

**Table 8:** The monthly average scores of assessment competent department in 2020 and 2021 (scores).

**Assessment competent department**	**2020**	**2021**
Medical record department	109.72	105.88
Quality control department	68.67	58.67
Department of medical administration	21.25	61.33
Outpatient department	19.17	6.33
Pharmacy department	10.17	9.42
Blood transfusion department	7.92	5.29
Prevention and health care department	3.33	9.67
Infection management department	1.75	3.75

**Table 9:** Evaluation results of 2021 annual scoring for physicians and medical technicians.

**Person in charge**	**Score**	**Evaluation results**
A	135.9	Unqualified
B	134.4	Unqualified
C	65.0	Warning
D	63.5	Warning

All personnel were qualified in the assessment in 2020, and all personnel in charge of assessment in 2021 were clinical department personnel of inpatient department. A, B, C, D are physicians and medical technicians.

## The characteristics of the assessment system

### The whole-process assessment

A whole-process quality management system for the entire diagnosis and treatment process starting from the initial visit to the eventual discharge of the patient was established. It consisted of various individual assessment indicators concerning all aspects of medical care, as well as clarification of their content, and their incorporation into the daily monitoring of the medical management department. With the adoption of the three-level structure-process-outcome theoretical model proposed by Avedis Donabedian, the management model was changed from one that relied excessively on end-point quality to a management model that focused on the combination of the results of medical structural quality, link quality and end-point quality process (7–9). In other words, a refined, scientific, and information-based whole-process quality control management model for healthcare quality and safety before, during, and after the event was established (10).

### Comprehensive assessment

A comprehensive quantitative assessment of the behavior and performance of doctors and other medical and technical staff was implemented. The defective score was adopted as the main method of assessment, and the reward score as an incentive measure. The score assessment was linked with the performance of clinical staff and their departments. Dynamic monitoring of the individuals and departments was conducted to continuously strengthen awareness of the need for quality healthcare and the practice of medicine following the law. The assessment was aimed at ensuring the regulation of the clinical treatment behavior of medical personnel, the elimination of medical safety hazards, and the insurance of the strict implementation of quality control measures. It enabled the medical personnel to be clearer about the code of medical conduct and to change their practice from passive to active observation of the code.

### Personalized assessment

The scorekeeping methods were divided into three categories, according to the characteristics of the different departments: the inpatient clinical department, the non-inpatient clinical department, and the medical and technical department staff. The assessment content and weighting were set according to the characteristics of the specialty, which improved the operability of the assessment. In addition, although the scorekeeping and penalties for residents in standardized training were the same as those of the hospital staff, there was a capped amount of penalty deductions for personalized assessment to maximize the fairness of the assessment. Meanwhile, diversity in the assessment could increase team effectiveness and performance (11).

### Information-based assessment

An assessment management system was established to improve the informatization of the assessment process. All points concerning scorekeeping and incentives were recorded in the medical information management system, and the system was able to automatically produce statistics. Staff could raise a query through the system to get a real-time understanding of their personal assessment points in scorekeeping, and they could then make an appeal to the lead department in the case of any objection. At the same time, the assessment results were entered and published in the medical information management system, which allowed for the extraction, management, disposal, and summarization of all data (12).

## Reflections and experience

By comparing the scores of the past 2 years, it can be found that the hospital's medical quality and safety evaluation system is running smoothly and effectively, and the average scores of doctors and medical technicians per month decreased as a whole. Practicing medicine according to law and medical insurance service management were not scored. The monthly score of medical record management, medical record writing and medical score system, medical ethics and practice management decreased, but the score was still very high, indicating that medical record management, medical record writing and medical score system management need to be further strengthened. Reasonable examination (medication, treatment, use of high-value consumables) and nosocomial infection management are also important manifestations of hospital management, which should be attached great importance to the rising score. The average monthly score per person in non-inpatient clinical departments and medical technology departments was lower than that in inpatient clinical departments. The overall decrease in the score of the surgical department indicates that the surgical line pays more attention to medical quality and safety than before. The medical record department, quality Control Department and medical Department of the competent departments scored the most points on average monthly for the examination objects, mainly because the medical record Department and quality Control Department supervised the most indicators, while the medical record Department was mainly because the medical records were not submitted in time. The annual scoring system of the hospital is mainly self-supervision. Third-party evaluation can be considered for auxiliary multi-mode and multi-dimensional evaluation, such as performance evaluation, hospital evaluation, German Medical Transparency Management System and Standards Committee certification, etc. However, external review is limited by cycle limit and post-supervision difficulty.

### It is important that the establishment of an assessment system is closely integrated with the policy

The viability of quality improvement depends on the effective functioning of key quality improvement systems such as operations, coordination, operational control, development, and policy (13). The evaluation standards were continuously revised and improved in the hospital in line with national and regional medical and health management requirements as well as the advanced practices of well-known hospitals, and, eventually, an annual healthcare quality and safety scorekeeping assessment system for doctors, medical, and technical staff that suited the actual situation of the hospital was established. The system now in place has improved the quality of healthcare in the hospital, and medical staff awareness of healthcare quality and the practice of medicine following the law has been strengthened.

It is essential that the assessment system is scientific and operable. (1) The three dimensions of structure, process, and results should all be taken into consideration in building a comprehensive evaluation indicator system focusing on process indicators with a result-oriented approach (14, 15). At the same time, a leading group for evaluation work should be established to regularly summarize and analyze the evaluation work, and continuously improve the evaluation system. (2) A full range of quantitative assessments should be implemented, and, with the inclusion of incentives and penalties, management compliance and self-management initiatives can be improved (16). A reward-based evaluation management system can be established, and management compliance and self-management initiative can be improved. (3) Strengthen the training of medical staff on medical quality knowledge, including the study of typical cases. Also, individual performance can improve the attention of the medical staff to the healthcare quality of the whole hospital as well as provide active participation in its management (17). This can change the concept of healthcare quality and safety in terms of ideological understanding. (4) When the assessment content and weighting is set according to the speciality, the operability of the assessment is improved, and the assessment itself is fairer. (5) Informatization in hospitals means a change from the traditional human supervision mode to an information-based supervision mode, from the statistical analysis of results to real-time monitoring of link quality. In addition, it implies a move from post-remediation and passive management to forward-looking prevention, control, and active management, and from on-site inspection and feedback to information-based real-time monitoring and feedback, supplemented by on-site inspection (18, 19). Such changes offer hospital staff an improved working environment and promote the scientific management of hospitals (20). (6) The new assessment system depends on the strong leadership of the department director. With the clarification that the department director has overall responsibility for the healthcare quality and safety of the department, management has the opportunity to reflect on its role, facilitate supervision and accountability, address healthcare quality problems, and avoid management chaos (21, 22).

## Conclusion

An annual scorekeeping system, involving doctors, medical, and technical staff, was established to assess the quality of healthcare and safety in our hospital. It is a process-driven, comprehensive, personalized, and information-based assessment system, which has succeeded in improving the quality of healthcare and operational efficiency of the hospital. While the new system has already shown its worth in practice, it still has some limitations in terms of its scope. First, since the patient is the service object in a hospital, and patient satisfaction can reflect the quality of medical services, performance assessment indicators need to also include patient satisfaction. In addition, although doctors, medical, and technical staff have the greatest impact on healthcare quality, the nursing staff also play an important role in this regard, and it is therefore recommended that they should also be included in the assessment system. Finally, whether these evaluation indicators are comprehensive enough and whether the framework is reasonable can be evaluated *via* the interview or questionnaire survey of hospital leaders, functional department managers, medical and medical technicians, and even patients. The FMEA analysis method can be used to evaluate and improve the system.

## Data availability statement

The original contributions presented in the study are included in the article/supplementary material, further inquiries can be directed to the corresponding author/s.

## Ethics statement

The studies involving human participants were reviewed and approved by The Sixth Affiliated Hospital of Guangxi Medical University Ethics Committee. The patients/participants provided their written informed consent to participate in this study.

## Author contributions

W-CX and G-ML: conception and design of the research. QL and J-WG: acquisition of data and statistical analysis. MG and G-ML: analysis and interpretation of the data and writing of the manuscript. Z-YZ and W-CX: critical revision of the manuscript for intellectual content. All authors have read and approved the final draft.

## Conflict of interest

The authors declare that the research was conducted in the absence of any commercial or financial relationships that could be construed as a potential conflict of interest.

## Publisher's note

All claims expressed in this article are solely those of the authors and do not necessarily represent those of their affiliated organizations, or those of the publisher, the editors and the reviewers. Any product that may be evaluated in this article, or claim that may be made by its manufacturer, is not guaranteed or endorsed by the publisher.
